# Thermal Infrared Imaging-Based Computational Psychophysiology for Psychometrics

**DOI:** 10.1155/2015/984353

**Published:** 2015-08-03

**Authors:** Daniela Cardone, Paola Pinti, Arcangelo Merla

**Affiliations:** Infrared Imaging Lab, Institute for Advanced Biomedical Technology (ITAB), Department of Neuroscience, Imaging and Clinical Sciences, University of Chieti-Pescara, Via Luigi Polacchi 11, 66013 Chieti, Italy

## Abstract

Thermal infrared imaging has been proposed as a potential system for the computational assessment of human autonomic nervous activity and psychophysiological states in a contactless and noninvasive way. Through bioheat modeling of facial thermal imagery, several vital signs can be extracted, including localized blood perfusion, cardiac pulse, breath rate, and sudomotor response, since all these parameters impact the cutaneous temperature. The obtained physiological information could then be used to draw inferences about a variety of psychophysiological or affective states, as proved by the increasing number of psychophysiological studies using thermal infrared imaging. This paper presents therefore a review of the principal achievements of thermal infrared imaging in computational physiology with regard to its capability of monitoring psychophysiological activity.

## 1. Introduction

Understanding affective and psychophysiological states of a conversational interlocutor is fundamental for setting a proper communication, establishing social and affective ties, choosing social strategies, and maintaining a contingent interaction. Such understanding and the quantitative assessment of psychophysiological states represent one of the major challenges in applied psychophysiology and, more recently, one of the major issues in human-machine or human-artificial agent interaction.

In fact, a common key requirement for all typologies of the human-artificial agent interaction is to set up a contingent interaction, with the agent being capable of not only reacting to human actions, but also (or should) reacting in ways that are congruent with the emotional and psychophysiological state of the human user or interlocutor [1, 2].

Conventional approaches for assessing affective and psychophysiological states are based on the measurements of several physiological parameters expressing autonomic nervous system (ANS) activity, like skin sympathetic response (SSR), hand palm temperature, heart rate and/or breath modulations, peripheral vascular tone, facial expression, posture, gaze, and electromyography activity [3–5]. Apart from the assessment of facial expression, monitoring these parameters usually requires the use of contact sensors attached to the subject. More recently, some of them are monitored through watch-like or wireless devices.

In order to exceed limitations derived from the use of contact sensors, computational psychophysiology based on imaging approach can be recommended.

To this goal, thermal infrared (IR) imaging has been proposed as a potential solution for noninvasive and ecological recording of ANS activity [6]. Thermal imaging allows the contactless and noninvasive recording of the cutaneous temperature through the measurement of the spontaneous thermal irradiation of the body [7]. The psychophysiological activity can thus be assessed through its thermal effects recorded by thermal IR imaging. In fact, skin temperature is modulated by the ANS activity, which in turn regulates the cutaneous blood perfusion, the local tissue metabolism, and the sudomotor response [8–17]. Since the face is naturally exposed to social communication and interaction, thermal imaging for psychophysiology is generally performed by imaging the subject's face. Given the proper choice of IR imaging systems, optics, and solutions for tracking the regions of interest, it is possible to avoid any behavioral restriction of the subject [18, 19]. This possibility is particularly important, for example, in the developmental psychology or human-artificial agent interaction fields.

This paper reviews the state of the art in the field of thermal IR imaging-based computational physiology. The general intent of the paper is to provide insights about its potentialities and limits for its use in quantitative psychophysiology.

## 2. Thermal Signatures of Psychophysiological Signals

Thermal signatures of a variety of psychophysiological signals have been identified. In particular, it has been demonstrated that, through bioheat transfer models, it is possible to compute at a distance the cardiac pulse, the breathing rate, the cutaneous blood perfusion rate, and the sudomotor response. This section summarizes the methods and the results for computational physiology based on thermal IR imaging.

### 2.1. Cardiac Pulse

**Figure 1:**
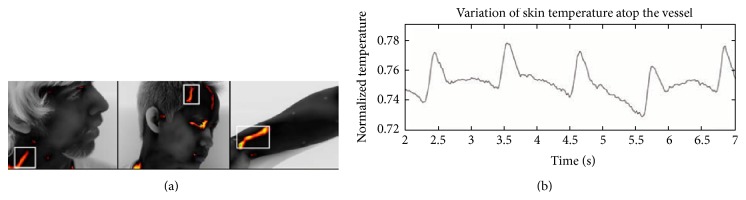
Pulse computation from thermal imaging data. (a) Collection point on the carotid arteriovenous complex, the frontotemporal region, and the wrist of the subject. (b) Temperature profile after removing frequency signals lower than 0.67 Hz (40 bmp) and higher than 1.67 Hz (100 bmp) (adapted from [9]).

Thermal IR imaging allows the computation of the cardiac pulse at a distance through the modeling of the pulsatile propagation of blood in the circulatory system [9, 20–23]. In fact, the heart contraction during the ventricular systole generates a pressure wave, which propagates through the arterial tree. The arterial pulse reflects the heart activity thus providing a measure of cardiac interbeat intervals, heart rate, and its variability [22]. The method presented by Garbey and colleagues [9] is based on the hypothesis that the temperature modulation due to pulsating blood flow produces the strongest variation on the temperature signal of a superficial vessel. The proposed model simulates the heat diffusion process on the skin originating from the core tissue and a major superficial blood vessel. They took into account noise effects due to the environment and instability in blood flow. Their simulation demonstrated that the skin temperature waveform is directly analogous to the pulse waveform, except for its smoothed, shifted, and noisy shape because of the diffusion process. The method proposed by Garbey and colleagues [9] for computing heart rate is based on the information contained in the thermal signal emitted from major superficial vessels and recorded through a highly sensitive thermal imaging system. To compute the frequency of modulation (pulse), the authors extract a line-based region along the vessel. Then, they apply fast Fourier transform (FFT) to individual points along this line of interest, to capitalize on the pulse's thermal propagation effect. Finally, they use an adaptive estimation function on the average FFT outcome to quantify the pulse (Figure 1). Experiments on a data set of 34 subjects compared the pulse computed from the thermal signal analysis method to concomitant ground-truth measurements obtained through a standard contact sensor (piezoelectric transducer). The performance of the method ranges from 88.52% to 90.33% depending on the clarity of the vessel's thermal imprint. Sun et al. [20] applied the same method but working at 90 degrees across the direction of the target vessel. An extension of the abovementioned methods has been realized by Bourlai et al. [21]. They applied these two methods on an automatic tracked region of interest (ROI) and added noise reduction through a two-stage algorithm that discards problematic frames as a result of bad tracking. The new method was tested on 12 subjects and reduced the instantaneous measurement error from 10.5% to 7.8%, while it improved mean accuracy from 88.6% to 95.3%.

More recently, Farag et al. [22, 23] proposed an automatic method to determine arterial pulse waveforms through the use of thermal imaging. This method is based on the hypothesis of the quasiperiodic thermal pattern on the skin due to the arterial pulse to automatically detect the areas surrounding superficial arteries. Multiscale decomposition models, such as wavelet decomposition, are applied to each thermal image to extract those scales containing most of the arterial pulse information. The influence of irrelevant noise is thus minimized and the arterial waveform recovery is more accurate. The more coarse scales are used to track the region of interest (ROI). The finer scales are used to compute the arterial pulse through the periodicity detection (PD) algorithm: a region of measurement (ROM) is chosen within each ROI and different ROM configurations are tested (size, orientation, scale, and location); for each tested ROM, continuous wavelet analysis is run to remove high frequency noise and to extract arterial pulses structures; maxima are calculated from the resulting waveform which in turn correspond to the systolic peaks (used to compute heart rate, beat to beat, and heart rate variability). The PD algorithm individuates the optimal ROM in terms of the periodicity of the waveform and of its relevance to the true arterial pulse propagation. Validation of the method on 8 subjects showed perfect matching with oximeter data [23].

### 2.2. Breathing Rate

**Figure 2:**
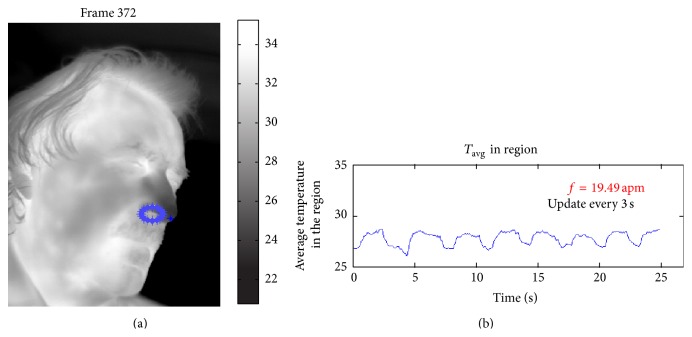
Thermal imaging data. (a) Thermal image showing the thermal track of the airflow. (b) Raw temperature versus time profile for a region of interest close to the nose tip.

Breathing consists of inspiration and expiration cycles during which heat exchanges occur between airflows and nostrils. These exchanges create a periodic or quasiperiodic thermal signal in the proximity of the nostrils that oscillates between high (expiration) and low (inspiration) values (Figure 2). Thermal imaging can capture this phenomenon at a distance, achieving an accuracy of 96.43% [8].

In conventional respiratory studies, a thermistor is usually positioned near the nostrils to capture this phenomenon and produce a representative breath signal [24].

Thermal imaging acts therefore as a virtual thermistor, since it captures the same process, but at a distance. The estimation of breathing rate through thermal imaging is very accurate as proved by comparison with respiratory signals taken with conventional sensors [25, 26]. From the work of Murthy et al. [25], a high degree of chance-corrected agreement (*κ* = 0.92) was found between the airflow monitored through thermal imaging and oronasal thermistors. Correlation coefficients between the thermally and mechanically (LifeShirt technology; see [26]) recorded breath rate signals resulted as high as 1 over a sample of 25 subjects, in both shallow, normal, and forced ventilations [26].

Lewis et al. [26] showed also the possibility of estimating the relative tidal volume from thermal imaging. The correlation coefficient between the thermal and meccanical recordings over the same sample was 0.90.

Statistical methods have also been proposed to compute the contactless breathing signature. The algorithm used by Murthy et al. [27] is based on the method of moments and Jeffrey's divergence measure. This method has been tested on 10 subjects leading to a mean accuracy of 92% compared with the respiratory belt data at the thorax.

Multiresolution analysis has been used as well [28, 29]. Fei and Pavlidis [29] extracted the breathing content from the mean temperature of the nostrils through wavelet analysis. They found a high degree of agreement between the thermally recovered breathing waveform and the corresponding thermistor one in 20 subjects. In the work of Chekmenev et al. [28] the nasal region is tracked over time and for each frame the ROI is decomposed and averaged at three different scales. Wavelet transform is then applied to the resulting signal. The scale that contains most of the breathing information is extracted and used to compute the breathing rate. This approach has been tested on 4 subjects and the results perfectly matched with the piezoelectric measure device signals.

Thermal IR imaging has been also used to retrieve breath-related thermal variations from nasal, ribcage, and abdomen regions of interest in children, both healthy and with respiratory pathology. The study proved that thermal IR imaging reliably acquires time-aligned nasal airflow and thoracoabdominal motion without relying on attached sensor performance and detects asynchronous breathing in pediatric patients [30].

Fei and colleagues [31] proposed a novel methodology to monitor sleep apnea through thermal imaging. The nostril region was segmented and tracked over time via a network of cooperating probabilistic trackers. Then, the mean thermal signal of the nostril region, carrying the breathing information, was analyzed through wavelet decomposition. The experimental set included 22 subjects (12 men and 10 women). The sleep-disordered incidents were detected by both thermal and standard polysomnographic methodologies. The high accuracy achieved confirmed the validity of the proposed approach for nonobtrusive clinical monitoring of sleep disorders [31].

### 2.3. Cutaneous Blood Perfusion Rate

**Figure 3:**
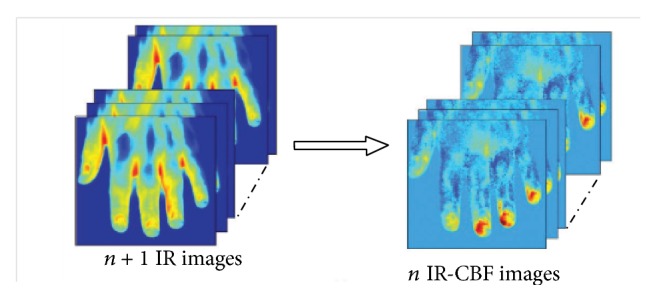
From the thermal IR image series to the cutaneous blood flow (CBF) images derived from thermal IR imagery. The series of IR images is converted into a series of IR-CBF images by applying computational models for bioheat exchange (adapted from [32]).

Bioheat transfer models permit the calculation of the cutaneous perfusion from high-resolution IR image series (Figure 3) [32, 33]. Pavlidis and Levine [33] even suggested to use cutaneous perfusion rate changes in the periorbital region as a performing channel for a new generation of deception detection systems, based on the flight-fight response of the inquired subject to sensitive questions. The models adopted are derived from previous works of Fujimasa et al. [34], Pavlidis and Levine [33], and Merla and colleagues [32]. According to these models, cutaneous temperature change over a short time is mainly due to the heat gain/loss via convection attributable to blood flow of subcutaneous blood vessels and the heat conducted by subcutaneous tissue.

The models show that the blood flow rate and the cutaneous blood flow depend mostly on the time-derivative of the cutaneous temperature and on the difference between the temperatures of the cutaneous layers and the inner tissues [32].

It has been demonstrated that it is therefore possible to transform raw thermal image series in cutaneous blood flow image series (Figure 3).

**Figure 4:**
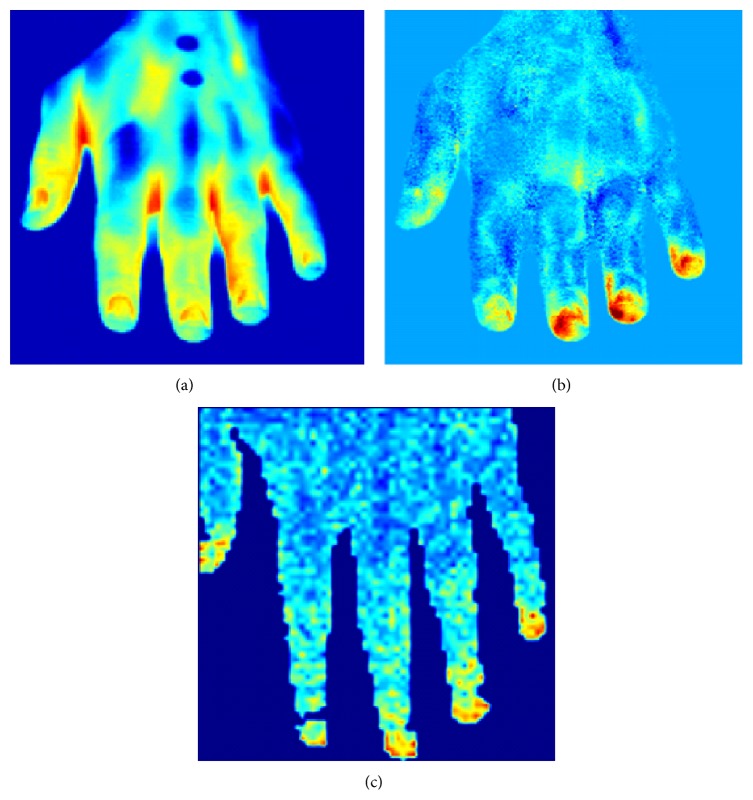
Computation of cutaneous blood perfusion from thermal image series. (a) Thermal image of healthy hand; (b) cutaneous perfusion computed from thermal imagery (in arbitrary units); (c) laser Doppler image (in arbitrary units). The overall distributions appear to be consistent, both images similarly showing the same high-perfusion and low-perfusion regions.

The method has been validated by comparison with laser Doppler imagery (Figure 4). Merla and colleagues showed that, in twenty healthy subjects, cutaneous blood flow values, simultaneously computed by thermal IR imagery and measured by laser Doppler imaging, linearly correlate (*R* = 0.85, Pearson Product Moment Correlation) [32]. The method has been applied in psychophysiology for deception detection [35] and emotion assessment [10].

### 2.4. Sudomotor Response

**Figure 5:**
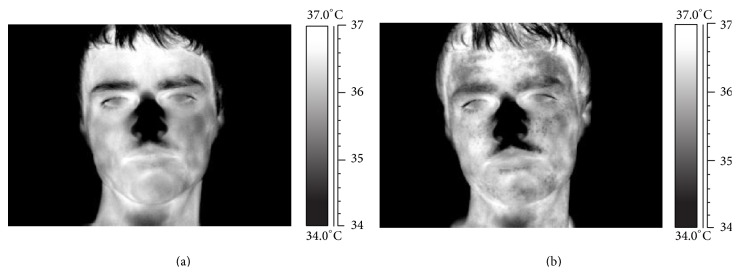
Emotional sweating and sudomotor response. The delivery of emotional pressure or stress stimulation (b) changes the rest of the (a) temperature distribution. The spotted dark signature is associated with the activity of the sweating glands.

Electrodermal responses have been among the most widely employed psychophysiological measures of autonomic nervous system activity. The Skin Conductance Response (SCR) and related measures, like galvanic skin response (GSR), have been shown to correlate with the number of active sweat glands, which activation can be easily visualized through facial thermal IR imaging by the appearance of cold dots over the thermal distribution of the face (Figure 5).

Concurrently to the palm area, strong sweat gland activation is manifested in the maxillary, perioral, and nose tip regions (Figure 5). Multiresolution analysis of the temperature changes reveals tonic (baseline and/or general) and phasic (event-related) components strongly correlated with GSR sympathetic constituents [12, 13, 16, 36]. For example, Pavlidis et al. [13] reported very high correlation coefficients between the GSR and the thermal measurement on the finger (*r*_MIN_ = 0.968) and on the perinasal region (*r*_MIN_ = 0.943). Moreover, wavelet analysis of thermal signals [12] revealed that the maxillary channel contains information about the sympathetic response almost as much as the GSR channel.

A number of studies suggest that the identification of active eccrine sweat glands by thermal imaging may have utility as a psychophysiological measure of sudomotor activity and may serve as a surrogate for the SCR when a contact method is either unavailable or undesirable [2, 6, 10, 12, 16, 36].

Recently, thermal IR imaging was used, together with standard GSR, to examine fear conditioning in posttraumatic stress disorder (PTSD) [37]. The authors examined fear processing in PTSD patients with mild symptoms and in individuals who did not develop symptoms, through the study of fear-conditioned response. The authors found that the analysis of facial thermal response during the conditioning paradigm performs like GSR to detect sympathetic responses associated with the disease.

### 2.5. Stress Response

An almost exclusive feature of thermal IR imaging in stress research is its noninvasiveness. Focused on professional drivers, a study of occupational ergonomics assessed mental workload using thermal IR imaging. Participants were exposed to simulator driving tasks both in the city and on the highway while cognitively challenged with a mental loading task (MLT). Compared with temperatures of the predriving session (baseline), significant differences were observed in the nose temperature across all conditions. The MLT seemed to have a defining effect on the temperature decrease of the nose, during the simulated city drive. No significant changes were observed on the forehead [38].

In a recent study, Pavlidis and colleagues [13] tried to quantify stress by measuring transient perspiratory responses on the perinasal area through thermal imaging. These responses proved to be sympathetically driven and, hence, a likely indicator of stress processes in the brain. The authors were able to monitor stress responses in the context of surgical training.

In another case and particularly in human-computer interaction field, Puri et al. [39] and Zhu et al. [40] used a Stroop task to exploit signs of frustration. Based on frontal regions, they observed that, compared with rest, stress increased blood volume into supraorbital vessels. Thermal IR imaging has also been used to assess affective training times by monitoring the cognitive load through facial temperature changes [41]. Learning proficiency patterns were based on an alphabet arithmetic task. Significant correlations, ranging from −0.88 to 0.96, were found between the nose tip temperature and the response time, accuracy, and the Modified Cooper Harper Scale ratings. Thermal IR thus represents a sensitive tool to assess learning and workload.

Engert et al. [15] explored the reliability of thermal IR imaging in the classical setting of human stress research. Thermal imprints were compared to established stress markers (heart rate, heart rate variability, finger temperature, alpha-amylase, and cortisol) in healthy subjects participating in two standard and well-established laboratory stress tests: the cold pressor test [42] and the trier social stress test [43]. Both tests showed evidence of thermal responses of several regions of the face. Although the thermal imprints and established stress marker outcome correlated weakly, the thermal responses correlated with stress-induced mood changes. On the contrary, the established stress markers did not correlate with stress-induced mood changes. These results suggest that thermal IR imaging provides an effective technique for the estimation of sympathetic activity in the field of stress research.

## 3. Discussion

Thermal IR imaging is a reliable method for ubiquitous and automatized monitoring of physiological activity. It provides a powerful and ecological tool for computational physiology. The reliability and validity of this method were proven by comparing data simultaneously recorded by thermal imaging and by golden standard methods, as piezoelectric pulse meter for pulse monitoring, piezoelectric thorax stripe for breathing monitoring or nasal thermistors, skin conductance, or galvanic skin response (GSR). As for the latter, studies have demonstrated that fIRI and GSR have a similar detection power [12, 13, 15, 35, 37]. Such results rely on the impressive advancement of the technology for thermal IR imaging. Modern devices ensure a high spatial resolution (up to 1280 × 1024 pixels with up to a few milliradiants in the field-of-view), high temporal resolution (full-frame frequency rate up to 150 Hz), and high thermal sensitivity (up to 15 mK at 30°C) in the spectral range 3÷5 *μ*m [44]. The commercial availability of 640 × 480 focal plane array of uncooled and stabilized sensors (spectral range 7.5÷13.0 *μ*m; full-frame frequency rate around 30 Hz; thermal sensitivity around 40 mK at 30°C) permits integrating this technology into automated systems for remote and automatic monitoring of physiological activity.

Real-time processing of thermal IR imaging data and data classification for psychophysiological applications is possible as the computational demand is not larger than that required for 640 × 480 pixels visible-band imaging data [2, 18, 44].

Thermal IR imaging has been indicated as a powerful tool to create, given the use of proper classification algorithms, an atlas of the thermal expression of psychophysiological responses [45, 46]. This would be based on the characterization of the thermal signal in facial regions of autonomic valence (nose or nose tip, perioral or maxillary areas, periorbital and supraorbital areas associated with the activity of the periocular and corrugator muscle and forehead), to monitor the modulation of the autonomic activity. Several studies have already shown the possibility of using thermal IR imaging in psychophysiology (see [2, 47] for reviews). These studies cover a number of fields, including developmental psychology and maternal empathy [48–50], social psychology [15, 51], and up to lie detection [52, 53].

However, several limitations exist for using thermal IR imaging in a real world. Because of the homeostasis, the cutaneous temperature is continuously adjusted to take into account the environmental conditions. Countermeasures must therefore be adopted to avoid attributing any psychological valence to pure thermoregulatory or acclimatization processes [2].

Also, despite the advantages offered by thermal IR imaging, it has to be taken into account that thermal signal development as a result of vascular change, perspiration, or muscular activity is rather slow with respect to other established techniques. Proper considerations should therefore be taken when monitoring thermal expression of psychophysiological activity.

Despite these limits, there is the concrete possibility of monitoring, in a realistic environment, at a distance and, unobtrusively, several physiological parameters and affective states. This opens the way for remote monitoring of the physiological state of individuals without requiring their collaboration and without interfering with their usual activities, thus suggesting the possibility of adding information of psychophysiological valence to behavioral or other typologies of investigation. One still unexplored but intriguing aspect is the study of possible correlation between individual thermal signatures and psychometric indexes, in order to assess, for example, whether given personality traits lead to interindividual differences in the facial thermal signature of autonomic activity or affective state or whether specific thermal expressions of specific personality or sociality traits exist. Of course, thermal IR imaging is not the first and unique attempt to explore these possibilities [54, 55], but thermal IR imaging seems to be one of the most ecological ones in this perspective. As such, thermal IR imaging provides an extraordinary opportunity to add physiological information to psychometric features, toward more robust classification of the individual's affective states, emotional responses, and profile.

A major issue that needs to be addressed for the practical application of thermal IR imaging in support of psychometrics concerns the adequacy of the method for identifying specific emotional or affective state at individual level. There are no specific studies available at the moment to answer this relevant question, which needs to be addressed by further research. A global limitation is derived from the fact that cutaneous thermal activity is intimately linked to the autonomic activity. The question therefore turns into “how specific and peculiar of each emotion are the autonomic responses and their thermal expression?” A definitive answer to this question is currently not available.
